# Investigation of risk factors for metachronous recurrence in patients with early gastric adenocarcinoma by miRNA–mRNA integral profiling

**DOI:** 10.1038/s41598-023-47000-3

**Published:** 2023-11-11

**Authors:** Ariki Nagashima, Kenichiro Okimoto, Ryo Nakagawa, Naoki Akizue, Tomoaki Matsumura, Hirotaka Oura, Ryuta Kojima, Chihiro Goto, Satsuki Takahashi, Ryosuke Horio, Akane Kurosugi, Tsubasa Ishikawa, Wataru Shiratori, Tatsuya Kaneko, Kengo Kanayama, Yuki Ohta, Takashi Taida, Keiko Saito, Tetsuhiro Chiba, Jun Kato, Naoya Kato

**Affiliations:** 1 Department of Gastroenterology, Graduate School of Medicine, Chiba University, Inohana 1-8-1, Chiba, 260-8670 Japan; 2 Division of Advanced Preventive Medicine, Graduate School of Medicine, Chiba university, 1-8-1, Inohana, Chiba, 260-8670 Japan

**Keywords:** Gastrointestinal cancer, Gene expression

## Abstract

The mechanism of metachronous recurrence (MR) after performing endoscopic treatment for early gastric adenocarcinoma (GAC) and eradicating *Helicobacter pylori* (*H. pylori*) is unknown. To elucidate the mechanism and risk factors of MR, we analyzed gene expression at multiple locations of the gastric mucosa. We selected each five patients with MR and without MR (control), after early GAC treatment and eradication of *H. pylori*. Mucosal tissue was collected from four sites in the stomach of each patient as biopsy specimens for mRNA sequencing, gene set enrichment analysis, and microRNA (miRNA) sequencing. We also performed correlation analysis and target prediction on pathways. As a result, endoscopically, the MR group had more intestinal metaplasia and enlarged folds. A total of 384 mRNAs presented changes in expression and 31 gene sets were enriched in the MR group. Immune-related pathways were enriched in the entire stomach, and the IFN-α response had the highest enrichment score. Additionally, 32 miRNAs revealed changes in their expression. Correlation analysis and target prediction with genes in the gene set of IFN-α response revealed that 10 miRNA–mRNA pairs presented a significant correlation. Immune-related pathways with miRNAs in the gastric mucosa after *H. pylori* eradication may be a risk factor for MR.

## Introduction

Gastric cancer remains one of the most life-threatening diseases, causing over a million deaths annually worldwide^1^. Most gastric cancers (approximately 90–95%) are adenocarcinomas (gastric adenocarcinoma; GAC)^2^. With the spread of endoscopic technology, GAC is often detected in its early stages and endoscopic treatment is widely available, providing results comparable to those of surgery while maintaining patient quality of life by preserving the stomach. However, gastric cancer recurs in 5.2–14% of patients after endoscopic treatment, which is a major clinical problem^3,4^.

The main cause of GAC is mucosal damage caused by chronic infection with *Helicobacter pylori (H. pylori)*, which is found in approximately 63% of gastric cancer cases^5,6^. The eradication of *H. pylori* reduces the risk of gastric carcinogenesis and prevents recurrence in patients after endoscopic resection^7,8^. Thus, endoscopic resection (endoscopic submucosal dissection and endoscopic mucosal resection) and eradication of *H. pylori* are generally performed in patients with early GAC with *H. pylori* infection^9^.

However, there are many cases of recurrence even after the eradication of *H. pylori* and endoscopic treatment of gastric cancer^3,4^. Metachronous recurrence (MR) of GAC can occur even when curative endoscopic resection for early GAC is performed and eradication of *H. pylori* is successful. Therefore, even after curative endoscopic resection of early GAC and eradication of *H. pylori*, almost all patients undergo regular post-treatment follow-up^9^. This causes patients to incur a large expense, and its contribution to the social cost also needs to be considered. Therefore, risk factors for MR after *H. pylori* eradication should be identified, and preventative methods should be developed.

In general, the risk of gastric carcinogenesis includes the degree of endoscopic atrophy in the gastric mucosa and the presence and extent of intestinal metaplasia^10,11^. This is also true for patients after *H. pylori* eradication and endoscopic treatment of early GAC^12^. However, the mechanism of this phenomenon has not yet been elucidated. Recently, genetic and epigenomic analyses have advanced with the widespread use of next-generation sequencers, and various reports on the molecular mechanisms of GAC have been published^13–15^.

MicroRNAs (miRNAs) are small non-coding RNAs that play an important role in post-transcriptional gene regulation in a variety of biological processes and have been reported to be involved in gastric carcinogenesis^16,17^. However, the gene expression changes, which occur in the gastric mucosa after *H. pylori* eradication and induce recurrence, along with miRNA changes, are unclear. Additionally, since the inflammation caused by *H. pylori* progresses heterogeneously in the gastric body, it is necessary to perform a wide range gene expression analysis of the gastric mucosa from each individual to understand the details of gene expression related to MR.

In this study, we performed an integrated analysis of microRNA (miRNA) and messenger RNA (mRNA) expression profiles of the gastric mucosa at multiple locations in patients with MR to elucidate the metachronous recurrence mechanism of GAC. The functions of the extracted genes were clarified by bioinformatics, and the expression profiles of miRNAs and mRNAs were compared with endoscopic findings and other clinical data to identify the regulatory factors of MR.

## Material and methods

### Patients

**Figure 1:**
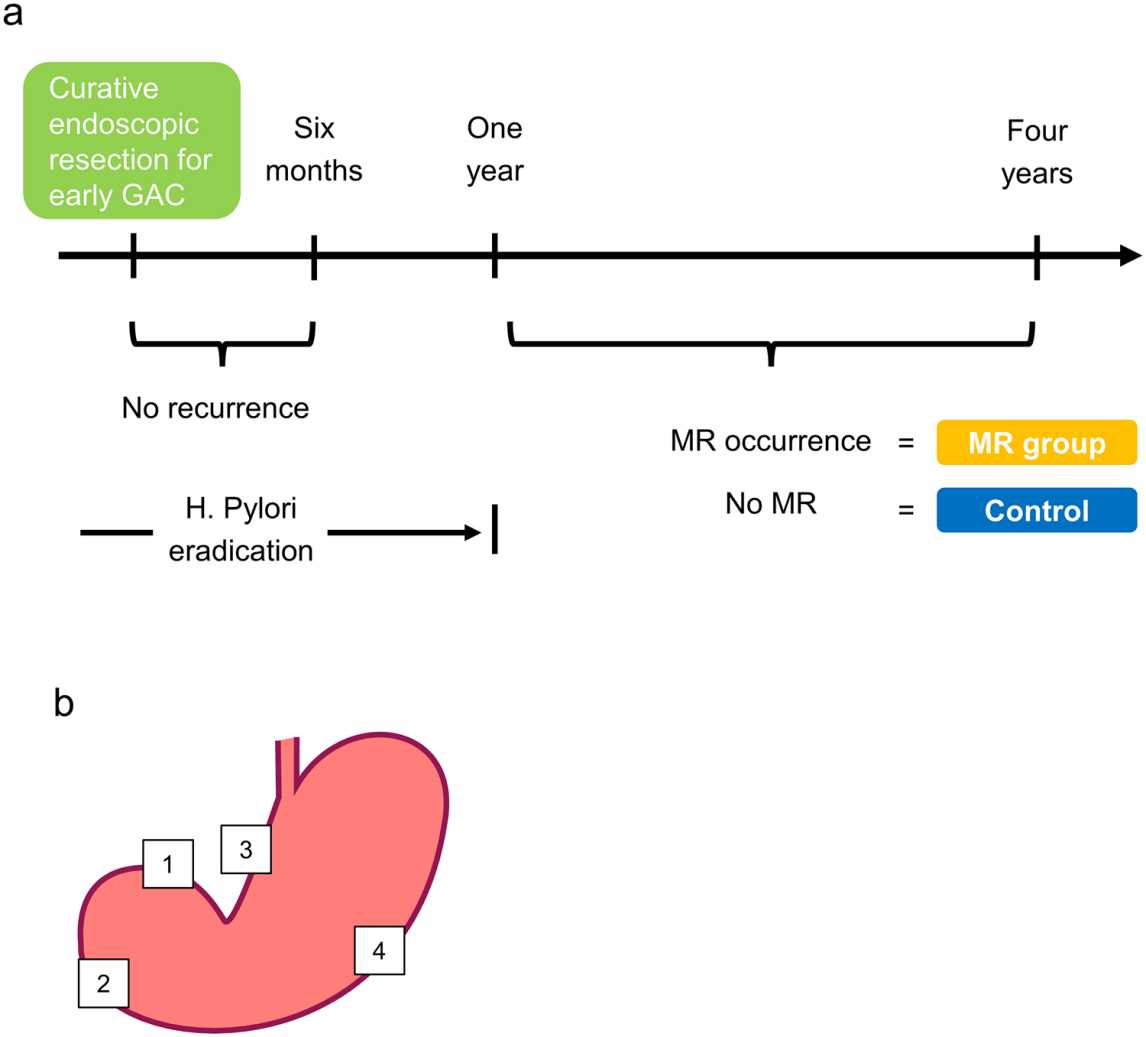
Study flow chart (**a**) Patient eligibility criteria. (**b**) Biopsies were taken from four different locations: 1. the lesser curvature of the antrum (LA), 2. the greater curvature of the antrum (GA), 3. the lesser curvature of the body (LB), and 4. the greater curvature of the body (GB).

Adults aged ≥ 20 years, who had previously undergone curative endoscopic resection for early GAC at our hospital, Chiba University Hospital, Japan, and underwent esophagogastroduodenoscopy at our hospital between November 1, 2019 and February 29, 2020, were deemed eligible and enrolled accordingly. Curative endoscopic resection is defined pathologically as differentiated mucosal cancer, without ulceration or with ulceration with a maximum diameter of less than 30 mm, without lymphatic or venous invasion, and with negative horizontal and vertical margins^9^. Patients who had *H. pylori* successfully eradicated 1 year after their first GAC treatment, had open-type gastric mucosal atrophy according to the Kimura-Takemoto classification^18^, and had never undergone upper gastrointestinal surgery were enrolled (Fig. 1a). From these, we selected five patients that did (MR group) and did not (control group) have MR after successfully eradicating *H. pylori*.

The MR group was defined as the group with patients who underwent curative endoscopic resection for GAC, presented no new GAC during endoscopic follow-up within six months, and presented newly developed GAC (differentiated cancer) in other parts of the stomach during endoscopic follow-up more than 12 months after treatment. In contrast, the control group was defined as the group with patients who did not have MR after more than four years of endoscopic follow-up after curative endoscopic treatment. GAC was finally diagnosed by pathological evaluation of endoscopically resected specimens.

The clinical characteristics, including age, sex, body mass index (BMI), drinking and smoking habits, the time interval between the first occurrence of GAC and biopsy, successful *H. pylori* eradication to biopsy, and history of GAC at the antrum or body of the patients, were investigated.

This study was reviewed and approved by the Institutional Review Board of the Chiba University School of Medicine. The purpose of this study was explained to all patients. Written and informed consent was obtained from all patients included in the study. All experiments were performed in accordance with relevant guidelines and regulations.

### Evaluating endoscopic findings

Modified grading scores for the Kyoto Classification of Gastritis were performed to evaluate the mucosal condition. The Kyoto classification score for gastritis is based on the sum of scores of the five endoscopic findings: Atrophy (A), intestinal metaplasia (IM), enlarged folds (H), nodularity (N), and diffuse redness (DR), which ranges from 0 to 8^19^. Although the IM score is usually determined by observation with white light imaging, we determined the IM score by observations with white light imaging and image-enhanced endoscopy, in a manner different from that of the Kyoto classification score. We summed the A, H, N, and DR scores and the modified IM score into a modified Kyoto classification score. This scoring was performed by three endoscopy experts who read the endoscopic images retrospectively.

### Biopsy sites

In each case, mucosal tissue was collected as biopsy specimens from four non-neoplastic gastric mucosal sites during endoscopic follow-up. These four sites were defined by the updated Sydney system^20^ and other previous reports^21^. Specimens were obtained from the lesser curvature of the antrum (LA) and from the greater curvature of the antrum (GA), both within 2–3 cm from the pylorus, the middle portion of the lesser curvature of the body between the cardia and angle (LB), and the middle portion of the greater curvature of the corpus (GB) (Fig. 1b).

### Extraction of RNA

After the biopsy, samples were placed in a microtube containing All Protect Tissue Reagent (Qiagen, Venlo, Netherlands), stabilized, and stored at -80 °C. Total RNA was extracted using the miRNeasy Micro Kit (Qiagen). Quality of total RNA was assessed before miRNA / mRNA sequencing by the protocol of the analysis service.

### Integral analysis for miRNA and mRNA

**Figure 2:**
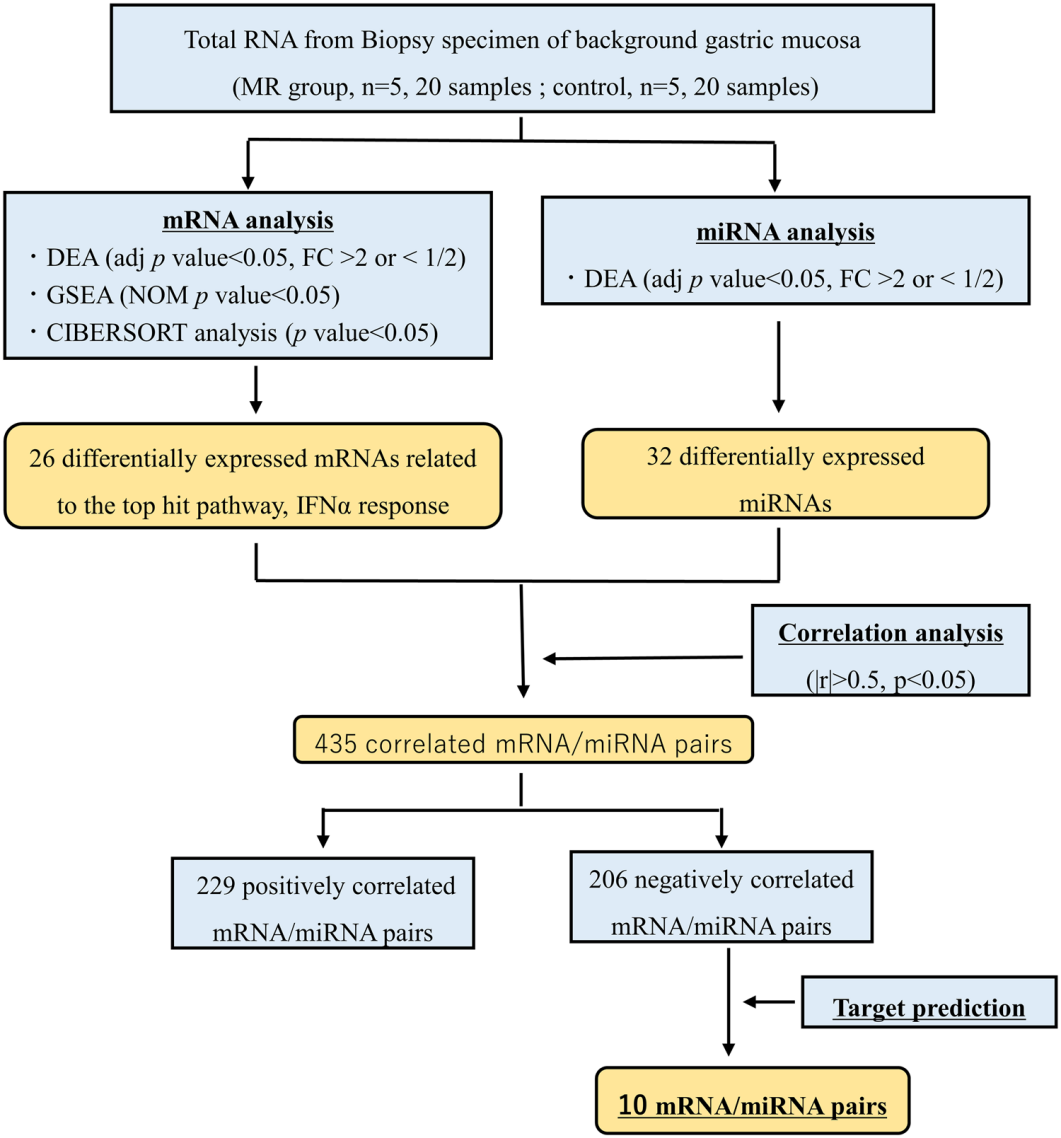
Analysis flowchart MR, metachronous recurrence; DEA, differential expression analysis; GSEA, gene set enrichment analysis; NOM *p* val, nominal *p*-value.

The extracted total RNA was sequenced for miRNA using the DNBSEQ-G400 (MGI Tech Co., Ltd, Shenzhen, CHINA) and mRNA using the Hiseq 2500 (Illumina, San Diego, CA, USA) by GENEWIZ Japan (Tokyo, JP) (Fig. 2). The Deseq2 package version 1.34.0 was used for expression analysis, and the BiomaRt package was used for mRNA annotation^22^. Gene set enrichment analysis (GSEA) was used for functional analysis of mRNAs, with the hallmark gene sets from the Molecular Signature Database (MSigDB) 7.4^23^. TargetScan 8.0 was used for miRNA target prediction^24^. Gene expression information was annotated to the predicted miRNAs and target genes, and the correlation between them was calculated. All authors had access to the study data and had reviewed and approved the final manuscript.

### CIBERSORT

CIBRESORT, a computational approach, was used to analyze the micro-immune environment within the gastric mucosa. Transcriptome data of all samples were analyzed according to the CIBRESORT analysis algorithm to evaluate the dynamics of the 22 kinds of immune cells (naïve B cells naive, memory B cells, plasma cells, CD8^+^ T cells, naïve CD4^+^ T cells, resting memory CD4^+^ T cells, activated memory CD4^+^ T cells, follicular helper T cells, regulatory T cells, gamma delta T cells, resting NK cells, activated NK cells, monocytes, M0 macrophages, M1 macrophages, M2 macrophages, resting dendritic cells, activated dendritic cells, resting mast cells, activated mast cells, eosinophils, and neutrophils) in the gastric mucosa^25^.

### Statistics

The Wald test was used for gene expression analysis, and the Pearson test was used for correlation analysis using R version 4.1.2^26^. The statistical program SPSS version 21.0 (SPSS Inc., Chicago, IL, USA) was used for statistical analysis of clinical characteristics and endoscopic findings. The chi-square test, Fisher's exact test, and Mann–Whitney U test were used, and a *p*-value of less than 0.05 was considered statistically significant.

## Results

### Clinical characteristics and endoscopic grading

A total of five patients with MR and five patients without MR (as controls) were enrolled in this study according to the criteria (Fig. 1a). There were no significant differences in the clinical background between the groups (Table 1). On evaluation by the modified grading scores for the Kyoto classification of gastritis, the MR group had higher scores of IM (intestinal metaplasia) and H (enlarged folds) and a higher overall score than the control (Supplementary Fig. 1).

**Table 1:** Clinical characteristics and endoscopic grading of patients.

	Control (*n* = 5)	MR group (*n* = 5)	*p*
Age, median(range) [years]	70 (66–80)	78 (68–79)	0.401
Male, *n* (%)	5 (100)	5 (100)	Matched
BMI, median(range) [kg/m^2^]	24.8 (22.1–30.1)	25.0 (20.8–25.9)	0.754
Alcohol consumption, *n* (%)	0	1 (20)	0.500
Smoking			
Current smoking, *n* (%)	1 (20)	0	0.500
Brinkman index, median(range)	600 (300–870)	800 (200–2500)	0.465
Time from first occurrence of GAC to biopsy, duration(range) [months]	95 (55–140)	63 (41–133)	0.076
Time from successful *H. pylori* eradication to biopsy, duration(range) [months]	95 (71–133)	62 (35–128)	0.251
History of antrum lesion, *n* (%)﻿	3 (60)	5 (100)	0.222
History of body lesion, *n* (%)	2 (40)	5 (100)	0.083
Modified Grading Scores for Kyoto Classification of Gastritis, median (range)			
A (Atrophy)	2 (2)	2 (2)	1.000
IM (Intestinal metaplasia)	0 (0–2)	2 (2)	0.017
H (Enlarged folds)	0 (0)	1 (0–1)	0.050
N (Nodularity)	0 (0)	0 (0)	1.000
DR (Diffuse redness)	1 (0–1)	1 (1–2)	0.093
Total	3 (2–5)	6 (5–7)	0.014

*MR*, Metachronous recurrence; *BMI*, body mass index; *GAC*, gastric adenocarcinoma.

### mRNA profiles of gastric mucosa from patients with MR

In the mRNA expression profiling, total RNAs of gastric mucosa from subjects were examined. All samples measured the A260 /A280 and RNA integrity number (RIN) for quality check [A260 / 280 (median (range)), 2.04 (1.91–2.10); RIN, 7.50 (4.90–9.10))] by the protocol of the analysis service.

FastQC were performed to check the quality of row sequence data by the analysis service. The peak of the average of quality scores was greater than 30, indicating that the sequence data of all samples were highly evaluated. Then, we filtered out from the total sequence data (60,612 rows) those rows that contained gene sequences unregistered in the ensemble gene ID and low-expressed genes for which the total value of all samples was less than 100 reads. The 20,584 mRNAs of sequence data obtained by this filtering were statistically analyzed.

A total of 384 mRNAs revealed expression changes in the comparison of the gastric mucosa at all four sites; 303 revealed an increased expression and 81 revealed a decreased expression in patients with MR compared to the control [adjusted *p*-value (adj-*p*) < 0.05, |Log2 fold change| (|LFC|) > 1].

In the greater curvature of the gastric body, 889 mRNAs were differentially expressed; 771 were upregulated and 118 were downregulated in patients with MR compared to the control. In the greater curvature of the antrum, 311 mRNAs revealed changes in their expression; 298 were upregulated and 13 were downregulated in patients with MR compared to the control. In the lesser curvature of the gastric body, three mRNAs revealed changes in their expression; two were upregulated and one was downregulated in patients with MR compared to the control. In the lesser curvature of the antrum, one mRNA was upregulated in patients with MR compared to that in controls.

**Figure 3:**
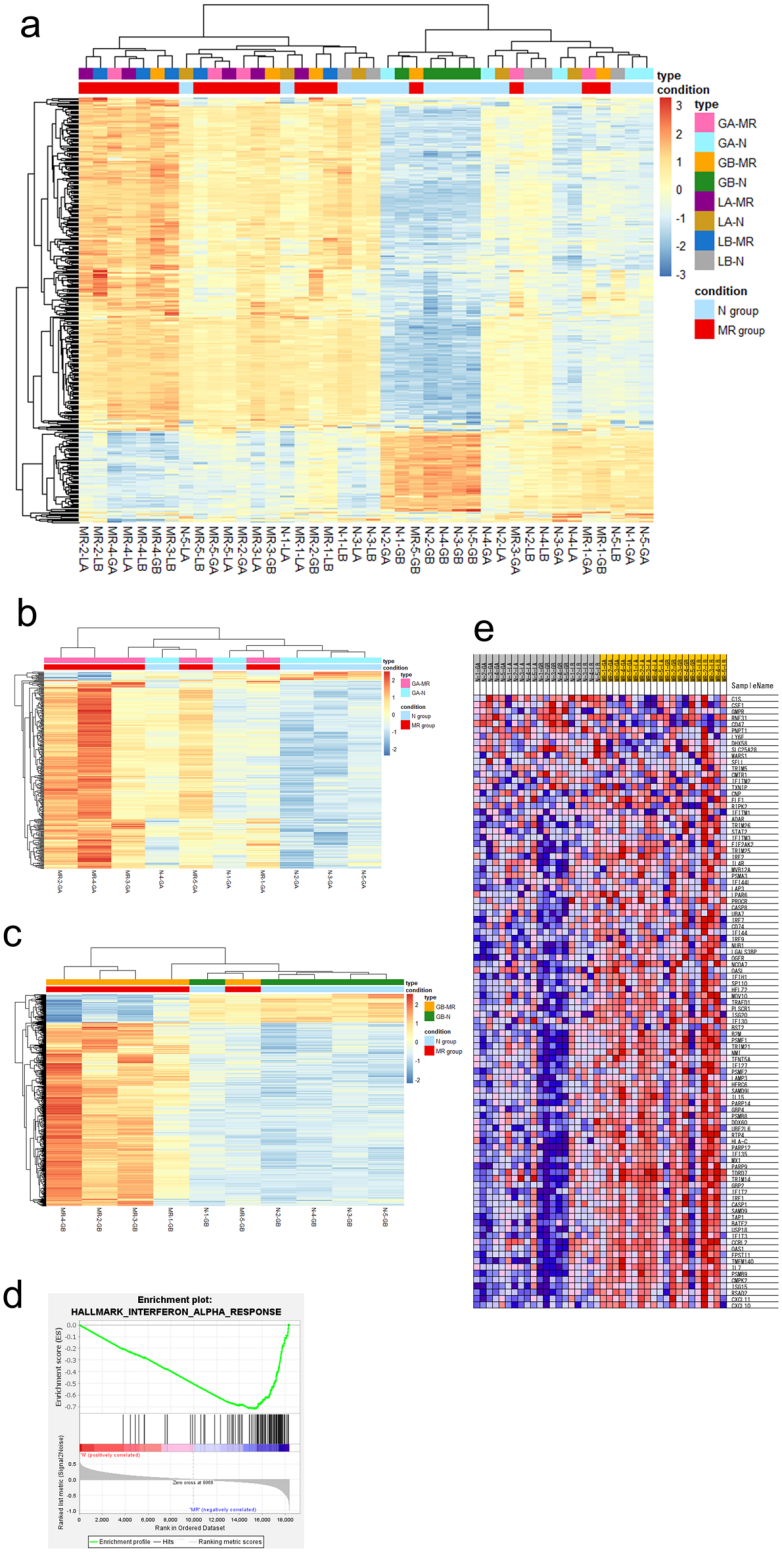
mRNA profiles and IFN-α response in GSEA (**a**-**c**) Heatmap of differentially expressed mRNAs by hierarchical clustering analysis (adjusted *p*-value < 0.05, |Log2 fold change|> 1). The following was analyzed: (**a**) all sites combined, (**b**) greater curvature of the body (GA), and (**c**) greater curvature of the body (GB). MR-1–5 are patients of the MR (metachronous recurrence) group. N-1–5 are patients of the control (non-MR) group. (**d**) Enrichment plot of IFN-α response at the analysis of all sites combined, which was the top hit at GSEA. (**e**) Expression of genes in the gene set of IFN-α response. Panels (**a**-**c**) were made using R version 4.1.2 and Pretty Heatmaps package. Panels (**d**-**e**) were generated using GSEA software (https://www.gsea-msigdb.org/gsea/index.jsp).

Hierarchical clustering analysis demonstrated that patients with MR and controls can be generally classified according to the expression patterns of 384 genes that revealed changes in their expression in all four gastric mucosae. Similarly, the expression patterns of 899 genes from mucosae in the greater curvature of the gastric body and 311 genes from mucosae in the greater curvature of the gastric antrum were used to classify patients with MR and controls (Fig. 3).

### Enrichment analysis for mRNA and predicted target genes

Gene set enrichment analysis (GSEA) was performed to elucidate the biological significance of the mRNA expression associated with metachronous recurrence of GAC. In the analysis of all sites, 31 gene sets, which were enriched in patients with MR, and four gene sets, which were dominant in the controls, were extracted (Table 2).

**Table 2:** Results of GSEA.

		All	LA	GA	LB	GB
Enriched in *N*-group						
Myogenesis	ES	0.4	–	0.41	–	0.41
	NOM *p*-val	0.003	–	< 0.001	–	< 0.001
Hedgehog signaling	ES	0.51	0.57	0.57	–	–
	NOM *p*-val	0.011	< 0.001	0.003	–	–
Epithelial mesenchymal transition	ES	–	0.3	–	0.37	–
	NOM *p*-val	–	0.03	–	0.017	–
Myc targets v2	ES	–	0.64	–	–	–
	NOM *p*-val	–	< 0.001	–	–	–
Enriched in MR-group						
Interferon alpha response	ES	− 0.72	− 0.69	− 0.67	− 0.66	− 0.68
	NOM *p*-val	< 0.001	< 0.001	< 0.001	< 0.001	< 0.001
Interferon gamma response	ES	− 0.6	− 0.59	− 0.54	− 0.51	− 0.61
	NOM *p*-val	< 0.001	< 0.001	< 0.001	< 0.001	< 0.001
Xenobiotic metabolism	ES	− 0.5	− 0.48	− 0.46	− 0.4	− 0.49
	NOM *p*-val	< 0.001	< 0.001	< 0.001	< 0.001	< 0.001
TNF-α signaling via NF-κB	ES	− 0.48	− 0.4	− 0.45	− 0.44	− 0.45
	NOM *p*-val	< 0.001	< 0.001	< 0.001	< 0.001	< 0.001
Complement	ES	− 0.46	− 0.39	− 0.44	− 0.39	− 0.51
	NOM *p*-val	< 0.001	< 0.001	< 0.001	< 0.001	< 0.001
Oxidative phosphorylation	ES	− 0.46	− 0.5	− 0.55	− 0.57	–
	NOM *p*-val	< 0.001	< 0.001	< 0.001	< 0.001	–
IL6 JAK STAT3 signaling	ES	− 0.49	− 0.47	− 0.45	− 0.41	− 0.52
	NOM *p*-val	< 0.001	< 0.001	< 0.001	< 0.001	< 0.001
Allograft rejection	ES	− 0.42	− 0.42	–	− 0.28	− 0.49
	NOM *p*-val	< 0.001	< 0.001	–	0.023	< 0.001
Cholesterol homeostasis	ES	− 0.47	–	− 0.48	− 0.49	− 0.41
	NOM *p*-val	< 0.001	–	< 0.001	< 0.001	0.038
Apoptosis	ES	− 0.42	− 0.35	− 0.36	− 0.33	− 0.47
	NOM *p*-val	< 0.001	0.027	< 0.001	< 0.001	< 0.001
Fatty acid metabolism	ES	− 0.39	− 0.44	− 0.42	− 0.32	–
	NOM *p*-val	< 0.001	< 0.001	< 0.001	< 0.001	–
P53 pathway	ES	− 0.37	–	− 0.38	− 0.34	− 0.36
	NOM *p*-val	< 0.001	–	< 0.001	< 0.001	0.019
Bile acid metabolism	ES	− 0.39	− 0.39	− 0.39	–	− 0.42
	NOM *p*-val	< 0.001	0.008	< 0.001	–	0.009
UV response up	ES	− 0.37	− 0.36	− 0.35	− 0.39	− 0.39
	NOM *p*-val	< 0.001	0.023	< 0.001	< 0.001	0.007
Hypoxia	ES	− 0.36	− 0.35	− 0.34	− 0.33	− 0.35
	NOM *p*-val	< 0.001	0.019	< 0.001	< 0.001	0.024
Inflammatory response	ES	− 0.34	− 0.35	− 0.32	–	− 0.38
	NOM *p*-val	< 0.001	0.017	0.006	–	0.005
Coagulation	ES	− 0.36	− 0.38	− 0.34	− 0.33	− 0.39
	NOM *p*-val	0.003	0.027	0.028	0.015	0.016
Heme metabolism	ES	− 0.34	− 0.38	− 0.36	− 0.35	–
	NOM *p*-val	0.009	0.003	< 0.001	< 0.001	–
Adipogenesis	ES	− 0.32	− 0.41	− 0.35	− 0.33	–
	NOM *p*-val	0.01	< 0.001	< 0.001	< 0.001	–
KRAS signaling up	ES	− 0.31	–	–	–	− 0.38
	NOM *p*-val	0.015	–	–	–	0.008
Glycolysis	ES	− 0.3	–	–	–	− 0.34
	NOM *p*-val	0.026	–	–	–	0.025
Mtorc1 signaling	ES	–	–	− 0.3	− 0.27	–
	NOM *p*-val	–	–	< 0.001	< 0.001	–
Estrogen response early	ES	–	–	–	− 0.28	− 0.34
	NOM *p*-val	–	–	–	0.024	0.037
Reactive oxygen species pathway	ES	− 0.37	–	–	–	–
	NOM *p*-val	0.079	–	–	–	–
Protein secretion	ES	–	–	− 0.35	–	–
	NOM *p*-val	–	–	0.009	–	–
KRAS signaling dn	ES	–	− 0.39	–	–	–
	NOM *p*-val	–	0.007	–	–	–
Pancreas beta cells	ES	–	–	–	–	− 0.54
	NOM *p*-val	–	–	–	–	0.006
Estrogen response late	ES	–	–	–	–	− 0.39
	NOM *p*-val	–	–	–	–	0.003
Spermatogenesis	ES	–	–	–	–	− 0.41
	NOM *p*-val	–	–	–	–	0.02
DNA repair	ES	–	–	–	− 0.28	–
	NOM *p*-val	–	–	–	0.033	–
Peroxisome	ES	–	–	–	− 0.3	–
	NOM *p*-val	–	–	–	0.038	–
Enriched in *N*-group at one site and in MR-group at another site						
Myc targets v1	N vs MR	–	N	–	–	MR
	ES	–	0.4	–	–	− 0.35
	NOM *p*-val	–	< 0.001	–	–	0.013
E2f. targets	N vs MR	MR	N	–	–	MR
	ES	− 0.3	0.38	–	–	− 0.52
	NOM *p*-val	0.033	< 0.001	–	–	< 0.001
G2m checkpoint	N vs MR	MR	N		MR	MR
	ES	− 0.31	0.37	–	− 0.25	− 0.55
	NOM *p*-val	0.018	< 0.001	–	0.032	< 0.001

GSEA was performed for each site (LA, GA, LB, and GB) and for all sites combined. Thirty-one gene sets enriched in patients with MR and four gene sets dominant in the controls were extracted. Three pathways were considerably enriched in both the controls and patients with MR, depending on the site.

*GSEA*, Gene set enrichment analysis; *LA*, lesser curvature of the antrum; *GA*, greater curvature of the antrum; *LB*, lesser curvature of the body; *GB*, greater curvature of the body; *MR*, metachronous recurrence; *ES*, enrichment score; NOM-*p* val, nominal *p*-value.

Similarly, GSEA was performed for all sites, and enrichment gene sets were extracted for patients with MR and controls. The interferon-alpha (IFN-α) response gene set had the highest enrichment score at all sites and was extracted as the top hit. The IFN-α response gene set contained 97 mRNAs, of which 26 presented significant expression changes in the expression variation analysis.

### miRNA profiles of gastric mucosa from patients with MR

FastQC were performed to check the quality of row sequence data by the analysis service. The peak of the average of quality scores was greater than 30, indicating that the sequence data of all samples were highly evaluated. Then, we filtered out from the total sequence data (3,553 rows) those rows that low-expressed genes for which the total value of all samples was less than 100 reads. The 545 rows of miRNA sequence data obtained by this filtering were statistically analyzed.

To understand the regulatory system of gene expression associated with metachronous recurrence of GAC, RNA sequencing was used to profile miRNA expression. In a comparison using the gastric mucosa at all four sites, a total of 32 miRNAs presented expression changes; 13 presented an increased expression and 19 presented a decreased expression in patients with MR compared to the control [adj-*p* < 0.05, |LFC|> 1]. In the greater curvature of the gastric body, 13 miRNAs were differentially expressed; four were upregulated and nine were downregulated in patients with MR compared to the control. In the greater curvature of the antrum, one miRNA was upregulated in patients with MR compared to the control. In the lesser curvature of the gastric body, no miRNA presented changes in the gene expression. In the lesser curvature of the antrum, 14 miRNAs presented gene expression changes; 9 were upregulated and 5 were downregulated in patients with MR compared to the control.

**Figure 4:**
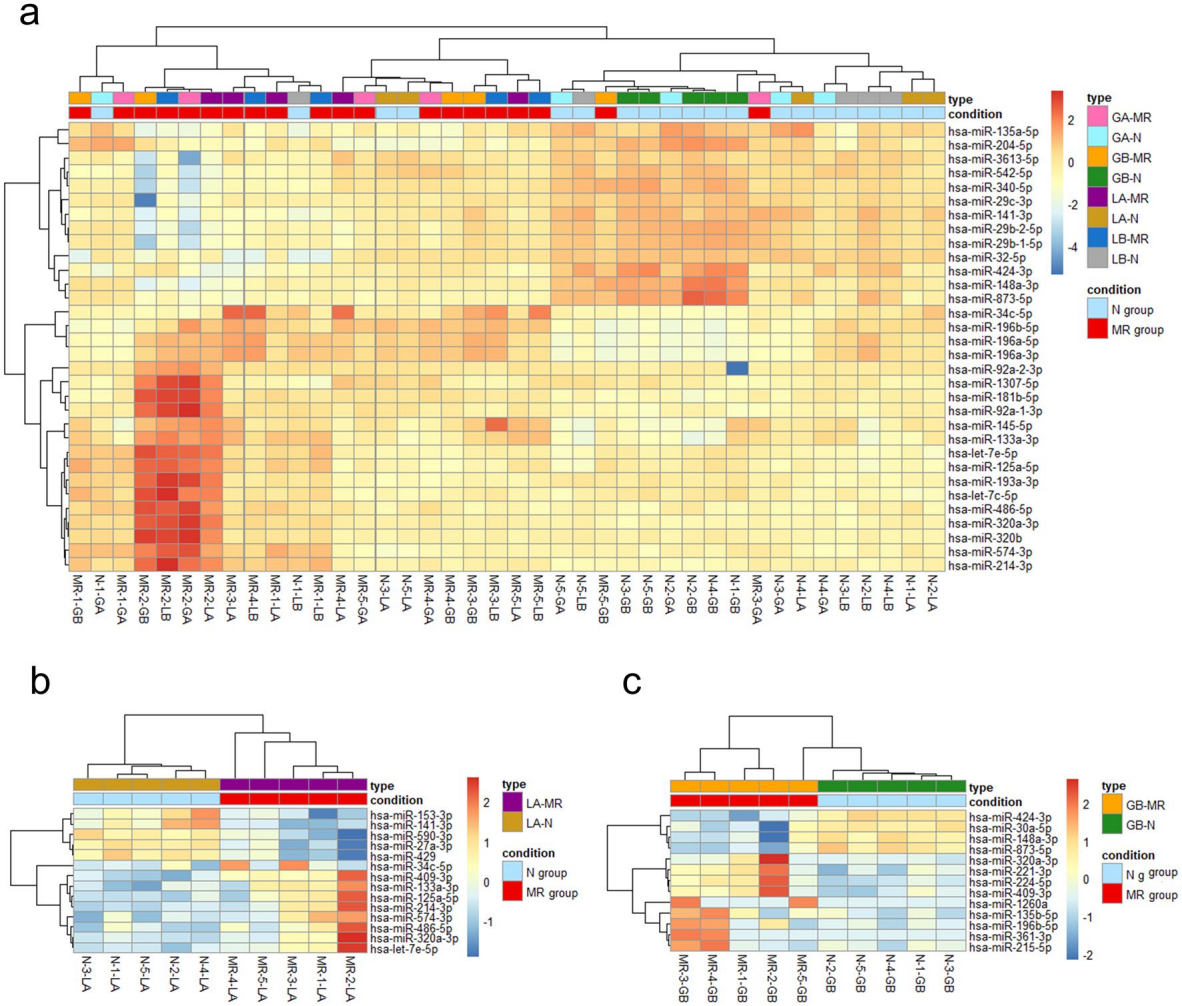
miRNA profiles Heatmap of differentially expressed miRNAs by hierarchical clustering analysis (adjusted *p*-value < 0.05, |Log2 fold change|> 1). Each of the following was analyzed: (**a**) all sites combined, (**b**) LA, and (**c**) GB. MR-1–5 are patients of the MR (metachronous recurrence) group, and N-1–5 are patients of the control (non-MR) group. Panels (**a**-**c**) were made using R version 4.1.2 and Pretty Heatmaps package. LA, Lesser curvature of the antrum; GA, greater curvature of the antrum; LB, lesser curvature of the body; GB, greater curvature of the body.

Hierarchical clustering analysis revealed that patients with MR and control can be generally classified according to the expression patterns of 32 miRNAs that presented expression changes in all four gastric mucosae (Fig. 4). Similarly, the expression patterns of 13 miRNAs in the greater curvature of the gastric body and 14 genes in the lesser curvature of the gastric antrum were used to classify patients with MR and controls.

### Correlation analysis of miRNAs presenting changes in expression to IFN-α response

**Figure 5:**
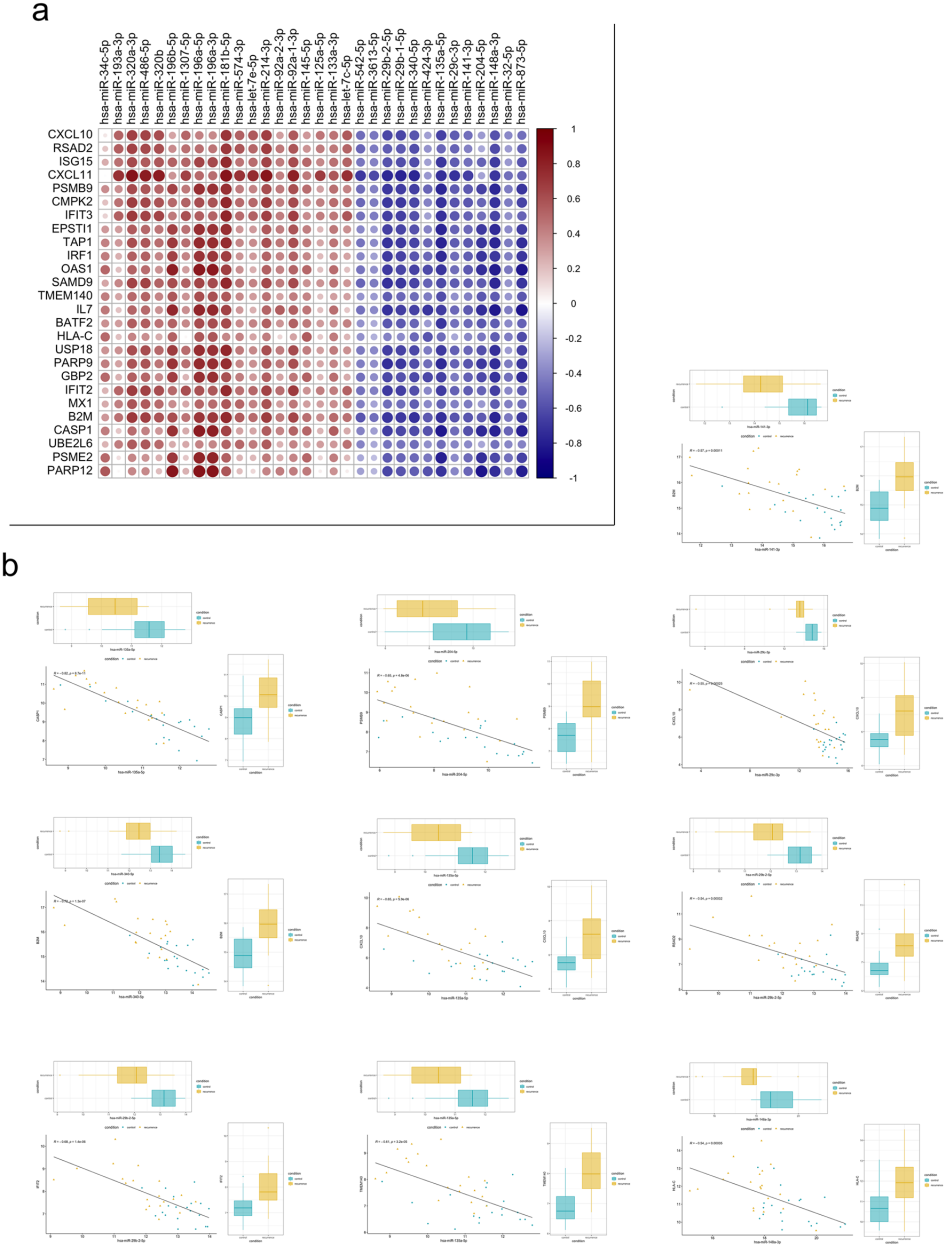
Correlation between mRNA and miRNA in IFN-α response (**a**) The correlation between 26 genes in the IFN-α response gene set and 32 differentially expressed miRNAs (|r|> 0.5, *p* < 0.05). (**b**) Correlation diagram of the mRNA-miRNA pairs that revealed a negative correlation and were predicted to be target gene pairs from the database: The central figure demonstrates miRNA and mRNA expression levels and regression line, and the figures above and to the right of the center demonstrate box plot of the expression levels of miRNA and mRNA in MR and control groups, respectively.

To elucidate the transcriptional regulation of the IFN-α response in gastric mucosa, the correlation between 26 genes in the IFN-α response gene set and 32 differentially expressed miRNAs was analyzed. Accordingly, 435 mRNA-miRNA pairs were correlated (|r|> 0.5, *p* < 0.05, Fig. 5a). Among these pairs, 206 presented a negative correlation and 229 presented a positive correlation between differentially expressed mRNAs and miRNAs. Of the 206 pairs, 10 pairs were predicted to be target gene pairs from the database and were negatively correlated with each other (Fig. 5b). The five pairs (miR-135a-5p/CASP1, miR-340-5p/B2M, miR-29b-2-5p/IFIT2, miR-204-5p/PSMB9, miR-135a-5p/CXCL10, and miR-135a-5p/TMEM140) presented particularly strong negative correlations (r < − 0.6, *p* < 0.05).

### Characteristics of immune microenvironment in the gastric mucosa from patients with MR

The microenvironment in background gastric mucosa of the MR group was analyzed using CIBERSORT to predict immunocytes associated with continued IFN-α/γ response. Seven of the 22 immune cell populations showed significant differences between the MR and control groups (*p* < 0.05, Supplementary Fig. 2). Four immunocytes (follicular helper T cells (*p* = 0.0013), gamma delta T cells (*p* = 0.0028), activated NK cells (*p* = 0.0054), and M1 macrophages (*p* = 0.0029)) were significantly increased in the MR group compared to the control group. Three immunocytes (CD8 + T cells (*p* = 0.01), regulatory T cells (*p* = 0.0072), and mast cells (*p* = 0.0003)) were significantly decreased in the MR group when compared with that in the control group.

## Discussion

We performed an integrated and comprehensive analysis of endoscopic findings and miRNA and mRNA profiles of the gastric mucosa of MR patients after eradication of *H. pylori*. It showed that the gastric mucosa of MR patients shows enlarged folds and specific mRNA and miRNA profiles within the tissue. Then, pathway analysis showed that immune-related pathway, such as IFN-α signaling pathway, were altered in the gastric mucosa of MR patients. Furthermore, miRNAs correlated with the expression of mRNAs in the IFN-α signaling pathway and were predicted to target these mRNAs.

First, inflammatory response such as IFN-α, IFN-γ, TNF-α, and IL-6 were elevated in MR patients. In previous reports, these inflammatory cytokines were also found in the blood of gastric cancer patients and were thought to be associated with chronic inflammation due to *H. pylori* infection.^27^. However, all patients with MR in this study had been eradicated of *H. pylori*. Therefore, it is very interesting that these inflammatory cytokine-associated molecules are upregulated in our patients with MR. Furthermore, endoscopic findings revealed enlargement of the greater curvature folds in patients with MR, even after *H. pylori* eradication. Enlarged folds are known to be a risk factor for gastric carcinogenesis, especially in patients with current *H. pylori* infection^28^, however usually improve shortly after *H. pylori* eradication^29^. Thus, enlarged folds after eradication of *H. pylori* suggests the possibility of a prolonged immune response in the gastric mucosa, which would normally be suppressed by eradication, and supports our mRNA profiling. It is an important issue for the further study to search for the cause of the inflammatory reaction in the gastric mucosa of MR patients after eradication of *H. pylori*. However, the aim of this study was to perform an integrated miRNA–mRNA analysis, and it was difficult to add sufficient samples for causal validation.

Representatively, we focused on the "IFN-α response" gene set, which was the top hit in GSEA. This gene set contained 27 differentially expressed mRNAs. The relationship of those 27 mRNAs to gastric cancer was discussed based on previous reports. Caspase-1 (CASP-1) is an inflammasome induced by type 1 interferon and regulates the secretion of cytokines and chemokines; IL-1β is a typical cytokine regulated by CASP-1, whose overexpression has been reported to induce gastric inflammation and carcinogenesis^30,31^. In addition, CASP-1 is reported to have both pro-inflammatory and regulatory effects in *H. pylori* infection^32^ and may be involved in the persistence of inflammation after eradication. Chemokine (C-X-C motif) ligand 10 (CXCL10) has been implicated in the promotion of gastric cancer invasion and carcinogenesis^33,34^. Moreover, CXCL10 is also reported to be induced by type I interferons^35^. Beta 2-microglobulin (B2M) binds to human leukocyte antigen (HLA) class 1, a transmembrane protein, and HLA expression in gastric cancer has been implicated in prognosis^36^.

To predict the biological functions of 32 MR-specific miRNAs, we analyzed the correlation between IFN-α-related genes and their expression. Interestingly, the expression of miRNAs and IFN-α-related genes revealed a strong correlation. Thus, we hypothesize that some of the MR-specific miRNAs are involved in the regulation of IFN-α-related genes. Particularly, miR-29b-2-5p, miR-135a-5p, miR-204-5p, and -340-5p have predicted target genes in IFN-α related genes and are strongly correlated with them. miR-135a-5p targets CASP1, CXCL10, and transmembrane protein 140 (TMEM140), which are known to be involved in the proliferation of gastric cancer cells and the expression of IFN-α. miR-135a-5p reportedly suppresses gastric cancer cell proliferation and migration in gastric cancer tissues^37,38^. miR-340-5p targets beta 2-microglobulin, albeit reportedly involved in the regulation of gastric cancer development and apoptosis^39,40^. These findings suggest that miRNAs with variable expression may be regulators of MR-induced chronic inflammation.

Additionally, along with these inflammation-related molecules, the p53-related gene cluster also presented changes, suggesting that oncogenic genetic changes also occur in the gastric mucosa of patients with MR.

On the other hand, the CIBERSORT analysis showed that some immunocytes in the gastric mucosa of the MR group fluctuated. It was reported that follicular helper T cells, one of the immunocyte increased in the MR group in our study, contributed to the continuation of interferon-γ-mediated chronic inflammation and carcinogenesis in the gastric mucosa infected with *H. pylori*^41^. Similarly, various associations between groups of immunocytes that exhibit fluctuations and gastric cancer have been reported^42–44^. While this study cannot clarify how those fluctuations are related to the enhanced IFN-α/γ response in the MR group and to gastric cancer recurrence, it is an important finding that elucidates the mechanism of gastric cancer recurrence.

In order to accurately elucidate the relationship of these IFN-α-related mRNAs and miRNAs to MR, a longitudinal analysis using a larger number of specimens is necessary. In this study, four gastric mucosal biopsies were taken from individuals, which pose a high patient burden. Therefore, it is desirable to verify these findings in a renewed study design.

In this study, we found enlargement of the greater curvature folds with upregulation of immune-related molecules even after eradication of *H. pylori* in MR patients. We hypothesize that these changes indicate ongoing inflammation of the gastric mucosa in MR patients, and that this ongoing inflammation induces carcinogenesis.

### Supplementary Information

Supplementary Figure 1.

Supplementary Figure 2.

## Data Availability

RNA-seq data for this study have been deposited in the Gene Expression Omnibus (GEO) under accession number GSE190459.
