# Supplementary figures and images for: Investigation of risk factors for metachronous recurrence in patients with early gastric adenocarcinoma by miRNA–mRNA integral profiling
Source: Sci Rep. 2023 Nov 11;13:19661. doi: 10.1038/s41598-023-47000-3 (PMC10640628; doi:10.1038/s41598-023-47000-3)

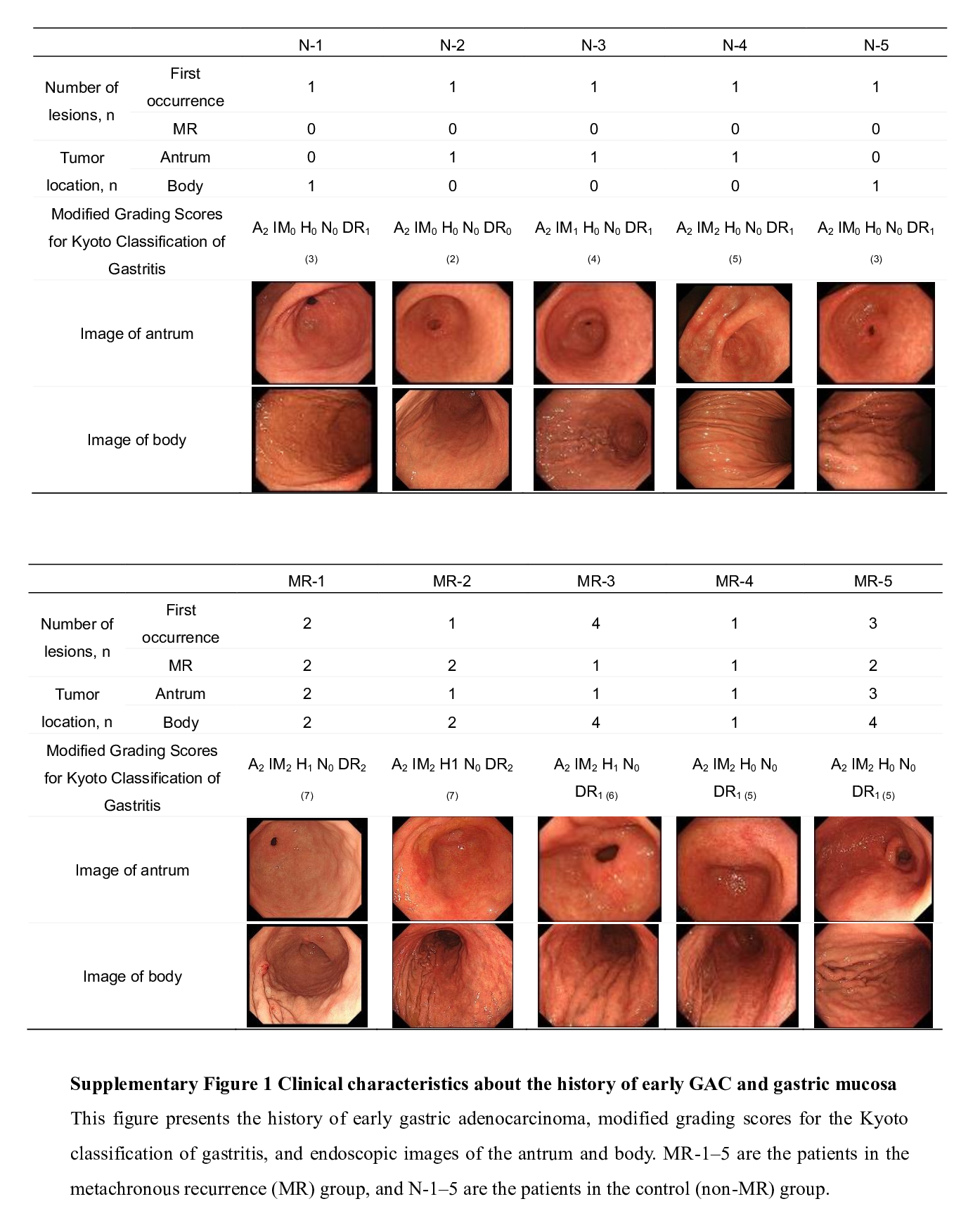

Supplement: Supplementary file 1 — Supplementary Figure 1. [file 41598_2023_47000_MOESM1_ESM.tiff]

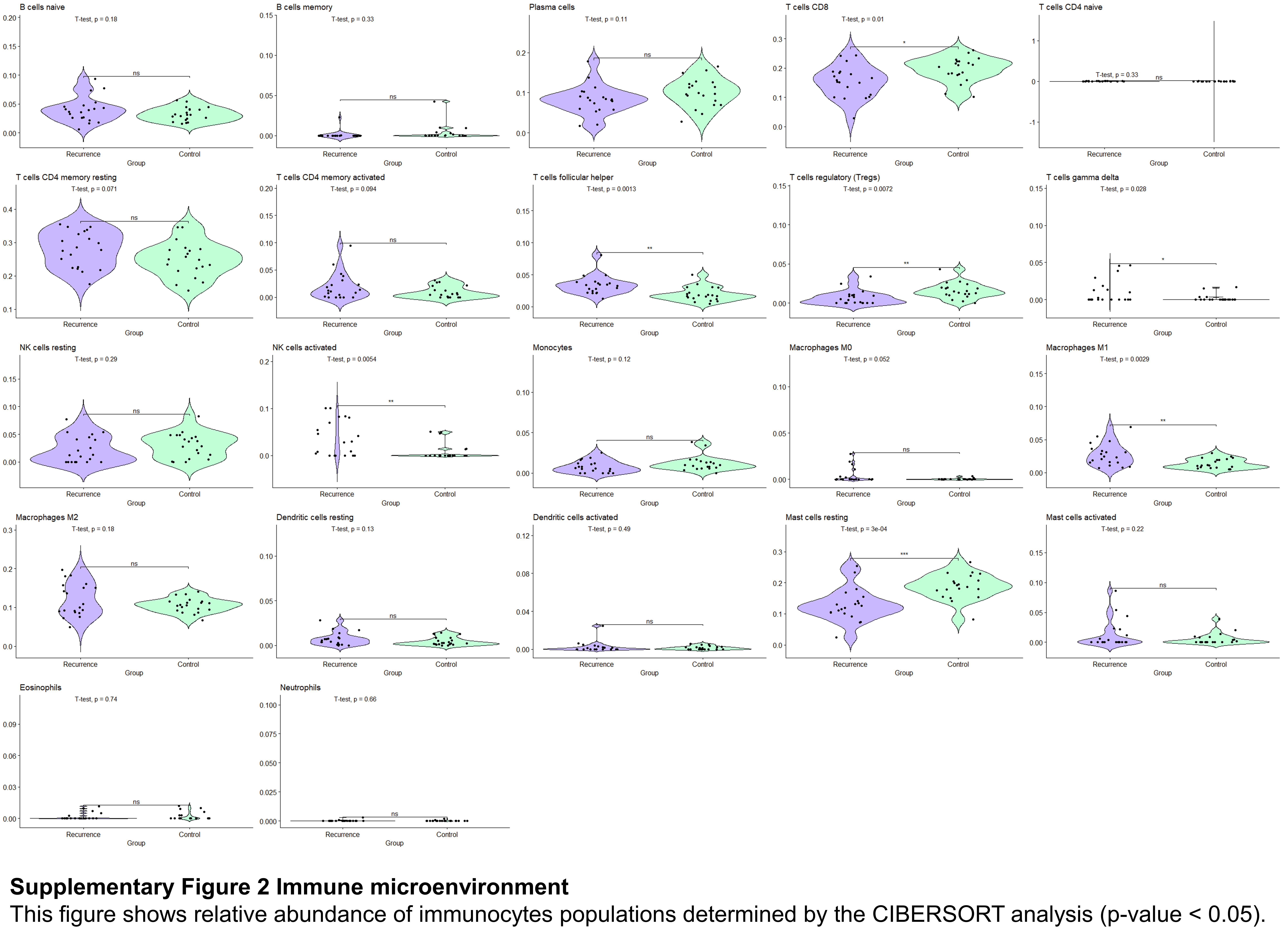

Supplement: Supplementary file 2 — Supplementary Figure 2. [file 41598_2023_47000_MOESM2_ESM.tif]
